# Incorporating patient reported outcomes (PROs) in gastro-intestinal (GI) cancer randomised controlled trials (RCTs): the need for adequate rationale and integrated reporting

**DOI:** 10.1186/1745-6215-12-S1-A76

**Published:** 2011-12-13

**Authors:** Rhiannon C Macefield, Angus GK McNair, Natalie S Blencowe, Sara T Brookes, Jane M Blazeby

**Affiliations:** 1 School of Social and Community Medicine, University of Bristol, Bristol, BS8 2PS, UK

## Objectives

Despite the widespread use of PROs in RCTs evidence suggests that results may not influence practice. Absence of an a priori rationale for measuring PROs and poor integration with trial clinical outcomes may contribute to this problem. This hypothesis was addressed by examining current reporting of PROs in GI cancer RCTs.

## Methods

A systematic review in MEDLINE, EMBASE and Cochrane databases searched for RCTs of radical treatments for GI cancer using validated PROMs, published between 2000 and 2009. Trials with a potential high risk of bias (Cochrane Collaboration tool) were excluded. Independent data extraction (3 reviewers) recorded the rationale for PRO measurement classifying this as 1) no rationale, 2) general rationale e.g. to examine QOL, 3) partial rationale e.g. hypothesis for a specific PRO domain or an expected direction of change or 4) complete rationale specifying a PRO domain *and* direction of change. Integrated reporting of clinical and PROs was investigated by examining whether trials reported PROs with or separately to clinical data, if PRO results were included in abstracts, and publication dates and journal impact factors where PRO and clinical results were published separately.

## Results

43 papers reporting PROs from 40 trials were included. Interventions were mostly chemotherapy (52.5%) and in colorectal cancer (77.5%). 16 (37.3%) papers did not report a PRO rationale, 14 (32.6%) gave general reasons, 6 (14.0%) a partial reason and 7 (16.3%) provided a detailed rationale describing hypothesised change in PRO domain and direction. Clinical and PROs were reported together in 30 papers (70.0%), in which PROs were typically a secondary trial endpoint (27/30, 90.0%). Of these, 11 had significant PRO results, with 10 (90.1%) reporting this in the abstract. 13 papers (30.3%) were separate reports of PRO data, supplementary to clinical findings. Median time between clinical and PRO publications was 21 months (range 5-51). The median journal impact factor for clinical and PRO papers was 15.6 and 6.3 respectively (p=0.03). Eight (61.5%) of the corresponding clinical papers did not report any PRO data and four (30.7%) made no indication that PROs had been measured despite the subsequent PRO publication.

## Conclusions

Few GI cancer RCTs provided a detailed rationale for measuring PROs and integration of clinical and PRO results was often poor. Standards for reporting PROs alongside clinical outcomes are required to improve clinical understanding and facilitate use of all trial data in decision-making.

